# Temporal and interaction dynamics of dengue cases, entomological and meteorological variables in Melaka, Malaysia: A multivariate time series analysis

**DOI:** 10.1371/journal.pone.0321273

**Published:** 2025-04-16

**Authors:** Shazelin Alipitchay, Muhammad Aswad Alias, Sharifah Nur Shahirah Syed Abdul Hamid, Rabizah Hamzah, Norain Mansor, Nurulhusna Ab. Hamid, Hidayatulfathi Othman

**Affiliations:** 1 Public Health Department, Melaka Health Department, Ministry of Health, Melaka, Malaysia; 2 Centre For Toxicology & Health Risk Studies (CORE), National University of Malaysia, Bangi, Malaysia,; 3 Institute for Medical Research, Selangor, Malaysia; Guizhou University, CHINA

## Abstract

The complex interaction between dengue cases, entomological and meteorological variables has posed challenges for decades. Validated and updated evidences are in need for enhancing surveillance and vector control of dengue program. This study explores the relationship between the variables in the long run and short-term dynamic in Melaka, Malaysia. A multivariate time series with the application of Johansen Cointegration Test and Vector Error Correction Model are carried out to validate the interaction among dengue cases, temperature, ovitrap index (OI) and sticky ovitrap index (SOI) data from 2020–2022. Cointegration vector validates existence of long-term relationship of which an inverse interaction between temperature and SOI with cases and a direct relationship of OI with cases. Short-term equilibrium displays a robust causality among variables. Interaction of case with case demonstrates positive coefficients at lags -3, -7, and -8. Interaction of SOI with case shows negative coefficients on SOI variable at lags -3 and -4 and positive coefficient on the case variable at lag -1. OI equation with OI variable shows unique interaction of negative coefficients on OI variable at lags -1, -3, and -4. However, it produced positive coefficient on OI variable at lag -9. Case equation reveals negative coefficient of temperature variable at lag -6. This study implies that the variables are linked in a long-term and stable relationship. In the context of public health, VECM is still a new methodology to capture such dynamicity and causality between the variables. In long term interaction, the study expressed the temporal pattern of dengue transmission, which is persistent, stable, and cyclical in nature. Failure to control epidemics resulting in the progression of succession of dengue cases in short term. The model predicts the utility and efficacy of sticky ovitraps acting as dual role; surveillance and control tool. Hence, there is a much broader scope for future directions in dengue control. The long-term equilibrium indicates the ovitrap index as a reliable predictor of dengue cases. Temperature is an overall excellent estimator of the meteorological parameter that has a direct impact on the development of dengue cases.

## Introduction

Dengue fever, recognised by the World Health Organisation as the most serious mosquito-borne viral disease, has increased in global occurrence by 30 times during the last five decades. This alarming trend disproportionately affects the Western Pacific and Southeast Asia, which account for 75% of global dengue incidence [1].

The complicated interaction between the Dengue virus (DENV), *Aedes* mosquitoes, human hosts, and environmental factors made disease prevention difficult. Due to the lack of viable treatments and immunisations, disease surveillance and vector control populations are still the core components of the current dengue preventive program [2].

A paradigm shift has been proposed: quick, sensitive, and comprehensive control approaches that prioritise extensive vector surveillance in addressing this growing threat [3]. However, establishing refined and targeted strategies necessitates a better knowledge of the complex interactions between host, environment, and entomological variables. Time series analysis is a promising method for revealing underlying trends and systemic patterns throughout time, providing for a more complete knowledge of the temporal dynamics and interconnections of crucial components. It is important to note that time-series analysis isn’t just about predicting the future; instead, it’s about understanding the past.

In the context of entomological studies in relation to dengue cases, immature mosquito or adult trapping indices are used to determine the level of infestation [4–6]. Methods used in entomological studies have evolved throughout the past decade. From the frequent use of comparative analysis or cross-sectional surveys [7–9] which can result in unclear conclusions and inadequately informed decision-making, to the use of considerably more complicated analysis, including time series [5,6,10]. The majority of these studies focus on the role of ovitraps as a surveillance tool, with no mention of the usage of sticky traps as vector control tools. Hence, investigation efforts should be shifted not only on entomological monitoring components but also on sticky traps as a control strategy. Therefore, the possibilities for future directions on dengue program are significantly greater.

The effects of meteorological variables on dengue cases are recently explored globally with the use of time series. A modelling study by Rachel Lowe et al. [11] highlights the role of climate variations, where droughts followed by heavy rainfall significantly drive outbreaks in Barbados. Another study shows how temperature influences vector and pathogen traits, ultimately shaping disease transmission dynamics. The study identifies a unimodal temperature-response relationship, with transmission peaking between 23–29°C and declining at lower and higher extremes [12]. While a study in Sri Lanka identifies significant time-lagged relationships between weekly rainfall, temperature, and dengue cases with the use of the El Niño-Southern Oscillation, known as ENSO-driven weather patterns [13]. A study in Bangkok integrates socio-economic, meteorological, and transport variables in relation to dengue risk, revealing how human mobility and urban connectivity amplify transmission [14]. Additionally, another study in Malaysia looked at the strongest correlation between the ground-based stations and the satellite-based Earth. It found that temperature, followed by rainfall and wind speed, is a useful meteorological variable for predicting dengue in future studies [15].

Two experimental studies on dengue cases were conducted in Malaysia to create a forecasting model by analyzing environmental, entomological, and epidemiological data series. Both studies highlighted the forecasting element. The first study showed only two significant variable data sets: the relation between entomological and environmental variables with the use of the autoregressive distributed lag (ADL) model [16]. The ovitraps data was used as the entomological variable. The previous week’s rainfall played a significant role in increasing the mosquito population, followed by maximum humidity and temperature. The second study was implemented in dengue-prone areas with the use of ovitraps and several mobile weather stations comprising rain gauges, temperature, and humidity data loggers located in the areas [17]. This is a follow-up study to address the cofounding factors and improve forecast accuracy. Significant findings were established among the three variables, with a forecasting accuracy of 85% for the specific area. Another study with the use of data from the two previous studies mentioned, proposed the installation of mobile rain gauges inside each dengue risk area to be integrated into the early warning system in order to generate a more precise prediction data [18].

This current study incorporates three variables: epidemiological, entomological, and meteorological data (temperature). Two sets of serial entomological data are being analyzed to measure the *Aedes* infestation level: ovitraps and sticky traps. This research attempts to elucidate the relationship between the variables in the long run and short term with the use of multivariate time series analysis. From this multivariate time series analysis, this study hopes to achieve and quantify the variables on their temporal associations, potential time lags, and dynamic interplays between all the variable data sets. It can offer insights into the cointegration of all variables, improve the understanding of the variables that have impact on the incidence of dengue cases, and guide specific targeted interventions and control measures in the area.

## Materials and methods

### Study area

This study is in Melaka Tengah, a high-density population with 1,673 people per square kilometer. The state consists of three districts: Melaka Tengah, Alor Gajah, and Jasin. The land covers an area of 1664 km^2^. It has a population of 985700 people. The state is situated in the southern part of Peninsular Malaysia. As of July 7, 2008, Malacca City, the capital of the state, has been recognized as a UNESCO World Heritage Site, attracting numerous tourists from around the globe. The city experiences an equatorial climate with high temperatures and humidity all year round. It receives heavy rainfall, especially between September and November. In the past five years, daytime temperatures have ranged from 31 to 33 °C (88–91 °F), while nighttime temperatures have averaged around 23 °C (73 °F).

### Epidemiological data

Epidemiological dataset is being accessed on 23 December 2023 for analysis. All human data are obtained retrospectively with the use of archived data (secondary data) which is a web base system under restricted authorization of Ministry of Health. All the data are de-identified as number only. Permission for access was obtained from the Public Health Division Director and with the review and approval of Medical Research and Ethics Committee (MREC), Ministry of Health Malaysia (MOH). Consent was initially obtained verbally during the investigation by Ministry of Health officers upon confirmation of diagnosis under the Prevention and Control of Infectious Diseases Act 1988. This was documented in investigation report.

Melaka recorded a total of 2843 dengue cases in 2020 with an incidence rate of 295 per 100,000 population, 611 dengue cases in 2021 with an incidence rate of 62.8 per 100,000 population, followed by a total of 665 dengue cases in 2022 with an incidence rate of 67 per 100,000 population [19]. Melaka Tengah accounted for the majority of reported dengue cases (70%). In Malaysia, dengue cases are reported as soon as a diagnosis is made and entered into an electronic notification web-based system. This system is then directly linked to a second web-based system called e-Dengue, which records all the profiles and locations of dengue cases. e-Dengue consists of data that has been validated epidemiologically and geocoded. The system also records control activity information. Thus, it is specifically designed to generate official daily report instantly. The data set on dengue cases included all registered cases that occurred between epidemiological week 1, 2020, and epidemiological week 52, 2022, spanning a total of 157 consecutive weeks.

### Entomological data

Relevant entomological datasets are being accessed on 26 December 2023. Two types of *Aedes* traps were employed: the conventional ovitrap and the autocidal trap [20–22]. They have been used as a standard entomological tool to survey the mosquito population. An ovitrap is a cylindrical plastic container with opaque black sides, which has a diameter of 7 cm and a height of 9 cm. A hardboard oviposition paddle of 10 cm × 3.0 cm × 2.5 cm is inserted into each ovitrap, with the rough surface facing upwards. The ovitrap was filled with tap water up to a height of 5.5 cm. A total of 60 ovitraps were installed within premises, semi-indoor, and outdoor per each location. They are placed on weekly basis at Station 1, located in Batu Berendam ecosystem, a public residential area. These containers were positioned there subsequent to acquiring consent from the homeowner. The second trap, known as the sticky trap or MyMAT (Malaysian Mosquito Autocidal Trap), is comprised of a 700-ml black plastic cylinder container. It is filled with approximately 600 ml of dechlorinated water and BTI (Bacillus thuringiensis) larvicide. An adhesive plastic strip, commonly known as a sticky card, was inserted into the container to capture the gravid adult mosquito during oviposition. The dechlorinated water, together with BTI and sticky cards, were replaced every two weeks. Then, the adult mosquitoes were extracted from the adhesive card using forceps, then identified using a 20X magnifying glass. The relevant data was subsequently recorded in the field during the trap inspections. A total of 200 sticky traps were strategically positioned similarly to ovitraps and were deployed at Station 2 within the Sungai Udang ecosystem, which is another public residential area. Both the ovitrap index and the sticky trap index can serve as estimators for the size of the *Aedes* population, the size of the larvae, and the size of the adults, respectively [20–22]. Sticky traps that capture adult female *Aedes* mosquitoes provide valuable insights into the likelihood of dengue transmission during an ongoing outbreak. The differences of several aspects of both traps are as summarized in Table 1 [20–22].

**Table 1:** Description of ovitraps and sticky traps used in study.

Aspect	Ovitraps	Sticky Traps
Design	Small black plastic containers filled with distilled water to attract female mosquitoes.	Incorporates adhesive surfaces to trap gravid mosquitoes attempting to enter.
Attractants	No infusion	Use Bacillus thuringiensis serotype israelensis, BTI (Vectobac WG) as attractant to attract adult mosquito
Size	Typically, small (0.5–2 liters) to mimic water-holding containers.	Varies in size but includes structural components to support adhesive mechanisms.
Durability	Requires regular maintenance (1x/weekly) (e.g., replacing water or infusion).	Periodic replacement of adhesive surfaces, generally lower maintenance.
Purpose	Monitors mosquito populations by collecting eggs & identifying larva species offering indirect measures of activity.	Directly captures adult mosquitoes, providing real-time data on abundance and infection risk.
Target Stage	Gravid female mosquitoes ready to oviposit eggs.	Adult mosquitoes, including potentially infected individuals, before oviposition.

**Fig 1:**
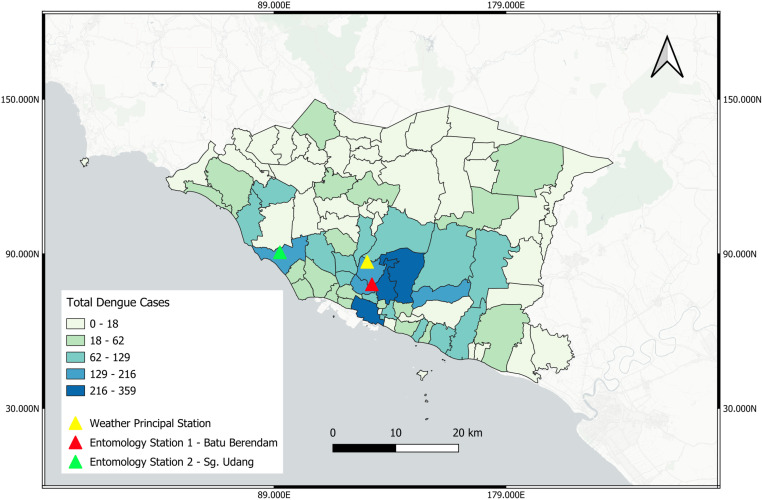
Statistical thematic map of Melaka with case aggregation, entomology stations and weather principal station.

A map of Melaka with aggregation of dengue cases, entomology stations and weather principal station are plotted with the use of statistical thematic map (Fig 1).

### Meteorological data

Temperature data set from 2020 up to 2022 was retrieved from Meteorological Department on 26 December 2023 and mean daily temperature are incorporated and aggregated into the study. These readings are taken from a principal station in Melaka.

### Data processing and analysis

R software 3.2.0 version is used to analyzed the time series data. The dataset comprises records of dengue cases (onset), ovitrap index, sticky trap index, and temperature observations spanning from 2020 to 2022. Each of the four data sets was graphed on its own panel, with a shared horizontal axis representing time in serial biweekly intervals.

### *Data process and analytical flow* (Fig 2)

**Fig 2:**
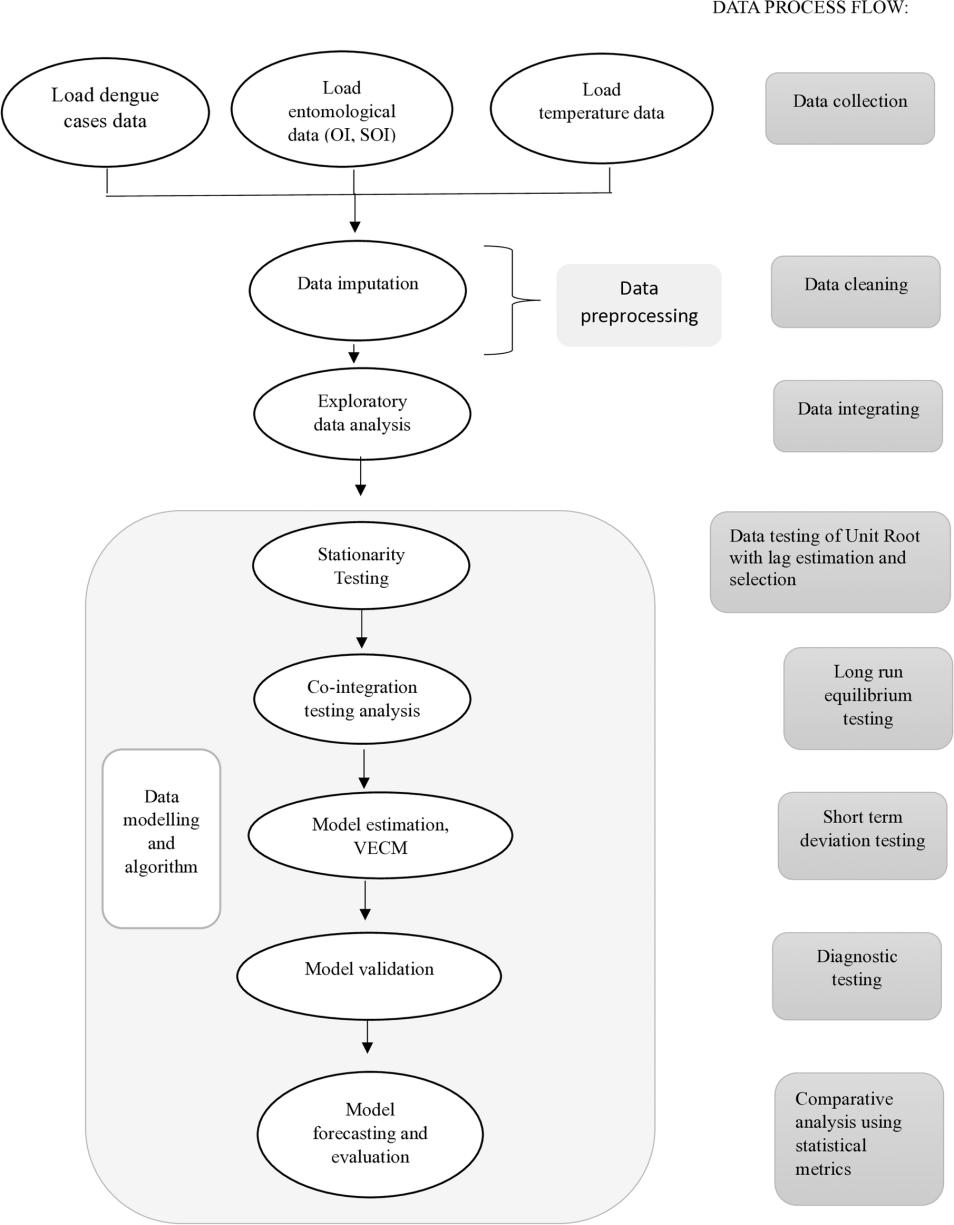
Illustrates and summarizes complex data testing.

With referring to Fig 2, all data are computed and loaded into four series. The flow is described accordingly;

1. ***Data cleaning***

This is in part of data preprocessing or also known technically as ‘Data imputation’. Missing values were treated appropriately using timeseries imputation R package, and the temporal resolution of the data was maintained.

2. ***Data integrating***

This comprised of preliminary exploratory data analysis and data transformation involving the process of combining and harmonizing data from multiple sources into a unified, coherent format of R package that can be put to use for various analytical and operational purposes.

3. ***Data testing of Unit Root with lag estimation and selection***

This consisted of stationarity testing of data series where ‘Augmented Dickey-Fuller’ test was employed. This is a crucial prerequisite for cointegration analysis and the application of the VECM. The assumptions of the ADF test;

i. The null hypothesis (H₀) assumes non-stationarity:

The ADF test assumes that the time series has a unit root (i.e., the series is non-stationary). Alternate Hypothesis (H₁): The series does not have a unit root and is stationary (mean-reverting).

ii. Linear relationship in the data:

The ADF test assumes the underlying relationship in the time series is linear (i.e., it does not test for stationarity in a nonlinear process). The data generating process is Autoregressive, AR. Hence, the ADF test is based on an autoregressive model where the current value of the series depends linearly on its previous values.

iii. Structural changes should be considered:

The ADF test may perform poorly if the time series contains structural breaks (e.g., sudden changes in mean or variance). Therefore, the test also assumes no structural breaks in the data, as unaccounted structural changes can bias the results. In such cases, more advanced tests like the Zivot-Andrew’s test should be used.

iv. Large sample size assumption:

The test assumes a reasonably large sample size for its properties to hold, ensuring reliable critical values.

v. Appropriate Lag Selection:

The test assumes a proper number of lags is chosen to control for autocorrelation in the residuals, ensuring valid inference. Lag selection can be automated using criteria like AIC (Akaike Information Criterion) [23]. A p-1 lag was applied in estimation, referring to the lag order of the error correction term. The ADF test was applied to both levels and first differences of the variables, and variables found to be non-stationary at levels were differenced to achieve stationarity. Lag effects were explicitly tested in the statistical models to capture the delayed relationships between dengue cases, climatic variables, and mosquito indices. Optimal lag lengths were determined using AIC, balancing model fit and complexity. A maximum lag of 10 was initially tested based on biological plausibility, considering the delayed effects of climatic factors like temperature on mosquito breeding cycles and dengue transmission. The 10-lag system specifically chosen via lag selection test using ‘Vector Autoregression, VAR’ package in R studio. Longer lags were tested randomly many times to capture the delayed impacts of climatic variables and vector indices on dengue cases. Each lag was assessed for statistical significance within the VECM. Base on the two critical elements; the ‘VAR’ package system and biological plausibility – balancing model fit and complexity, lag 10 was found to be the most optimal lag. Subsequently, it entails the need to use a specific model, ensuring that subsequent analysis yields valid and reliable results for forecasting or understanding the dynamics between these variables.

4. ***Long run equilibrium testing***

The cointegration of the variables was assessed using the Johansen cointegration test [24] of the eigenvalue approach. The test is to detect multiple cointegration time series which is appropriate for multivariate analysis time series [24] and is crucial to validate existence of stable long-term relationship. The key assumptions are;

i. The time series variables must be integrated of the same order.

For the Johansen test, it assumes that all the variables being tested are non-stationary to start with. They should all have similar behavior — meaning none of them are already stationary in their original form. If some variables are stationary from the beginning while others are non-stationary, the test won’t work because it is designed to detect long-term relationships only between variables of the same type. In short: all variables need to “start from the same place” (non-stationary) for the Johansen test to work. If one variable is already stable (stationary), a different method needs to be used.

ii. Vector error correction model (VECM) model assumption.

The Johansen test is based on a VECM model of order. The idea is that multiple time series can influence each other over time, and this relationship can be captured using this type of modelling. It is a way to explain how the current value of one variable depends not only on its own past values but also on the past values of other variables in the system. Hence, this test is to figure out if there is a long-term equilibrium relationship (cointegration) between the variables, even though they might move unpredictably in the short term. It examines if the variables “stick together” over the long run, despite short-term fluctuations.

iii. Linearity assumption

The Johansen test assumes a linear relationship between variables in the long-run equilibrium.

iv. The error terms follow a normal distribution

Residuals in the model should be normally distributed.

v. The rank of the cointegration matrix determines the number of cointegrating vectors.

The test examines the rank of the cointegration matrix, which determines how many cointegrating relationships exist. If rank, r=0, there is no cointegration, meaning no long-term equilibrium relationship. Hence, it cannot proceed with VECM. If 0<r<n (where n is the number of variables), there exist r cointegrating relationships as described partly in result section.

vi. Optimal lag selection

The Johansen test requires optimal lag selection as previously mentioned to capture the dynamics of the relationships without overfitting.

1. ***Short term deviation testing.***

The estimation of the model used and selected is done by looking into a series of cointegrations. If the series are not cointegrated, we can estimate the model via the vector autoregressive (VAR) function. If the series are cointegrated, the Vector Error Correction Model (VECM) is considered. This differs from the vector autoregressive (VAR) model in terms of the error correction term. VECM is a multivariate time series analysis that will compute the effect of *how the growth rate of a variable changes if one of the variables departs from its equilibrium value*. Hence, it allows both short-run and long-run coefficients, providing insights into equilibrium relationships and deviations from that equilibrium. VECM is able to capture the causal effect and determine the short- and long-term runs. A stable cointegration relationship ensures that the variables move together over the long run despite short-term fluctuations. Therefore, it is plausible and relevant in explaining the case-occurrence pathway.

6. ***Diagnostic testing***

For the model validation, diagnostic testing was performed. Residuals from the VECM were tested for autocorrelation, adequate lag selection, normality, and heteroscedasticity, ensuring model validity. Stability diagnostics confirmed that the model met the eigenvalue stability condition. These rigorous steps ensured that the statistical tests and model assumptions were met, providing a robust framework for analyzing the complex dynamics between dengue cases, climatic variables, and mosquito indices.

7. ***Comparative analysis***

Forecasting model based on VECM was captured with the use of comparative analysis to assess the forecasted biweekly onset values compared against biweekly average actual values. It is observed over a 12-week period, commencing from January 2023 until March 2023. Three primary statistical metrics, Mean Squared Error (MSE), Root Mean Square Error (RMSE) and Mean Absolute Percentage Error (MAPE) were employed to quantify the deviations between the VECM forecasts from the actual observed values.

The 12-week (6 period ahead in this case) timeframe provides a representative sample for short-term forecasting and reflects real-world applications, where public health responses require timely predictions. While 12 weeks may not capture long-term trends, it effectively demonstrates the model’s predictive capabilities in a critical window for dengue outbreaks. Longer period is highly potential to increase the uncertainty. Further projection for dengue cases would mostly relate to long term equilibrium, ignoring the possibility of short-term deviation. It is recommended to use deep machine learning however it does not relate to the objective of this study which is the crucial interaction of dynamicity and its real-world validation.

## Results

### Time plot data series

**Fig 3:**
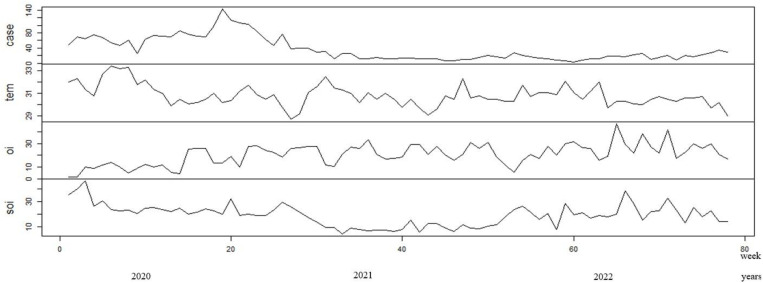
Four distinct panel of line graphs with case counts (dengue onsets), temperature (tem), ovitrap index (oi), and sticky ovitrap index (soi) readings on y -axis plotted against time in week from 0 up to 80 weeks (year from 2020 till 2022) on x axis, extracted from R.

The time series analysis of four distinct variables is depicted in the diagram (Fig 3): case counts (dengue onsets), temperature (tem), ovitrap index (oi), and sticky ovitrap index (soi). A common horizontal axis that represents time is shared by each variable, which is plotted in a distinct panel. The time plot is translated into biweekly serial data, resulting in 78 observations.

The case count exhibits significant fluctuation in the early stages but becomes rather consistent in later periods. In 2020, the number of dengue cases reached its greatest level of 1948 cases, with an incidence rate of 332.1 per 100,000 people. In comparison, there were 478 cases with an incidence rate of 80.4 in 2021, and 444 cases with an incidence rate of 73.8 in 2022. The temperature shows some fluctuation with no clear seasonal pattern discernible from this graph with minimum average temp of 28.7°C up to maximum average temperature of 33.4°C. The ovitrap index exhibits prominent deviations with peaks and troughs, while the sticky ovitrap index for adult mosquitoes demonstrates significant volatility with occasional spikes, potentially suggesting sudden increases in mosquito populations at specific intervals.

The number of cases varies in the beginning but reach to a fairly stable level later. With 1948 cases, the number of dengue cases peaked in 2020, with an incidence rate of 332.1 per 100,000. In contrast, the incidence rate of 478 cases in 2021 was 80.4, while the incidence rate of 444 cases in 2022 was 73.8. This graph also indicates that there is no obvious seasonal pattern in the temperature data series, with a minimum average temperature of 28.7°C and a maximum average temperature of 33.4°C. The sticky ovitrap index for adult mosquitoes shows considerable volatility with sporadic spikes, which may indicate abrupt increases in mosquito populations at particular periods. The ovitrap index shows notable deviations with peaks and troughs.

### Augmented Dickey Fuller, ADF unit root testing

The Augmented Dickey-Fuller (ADF) test successfully identified the existence of three non-stationarity data series, which were then transformed into stationary series using first difference. Guided by AIC, a lag length of 10 was determined to be optimal, providing a balance between model complexity and model fitting. This lag order is used for estimating Johansen cointegration and VECM tests.

### Cointegration analysis

#### Cointegration trace test statistics.

A 10-lag vector autoregression model was utilized to conduct the Johansen cointegration trace test on the variables. The findings show that there is one cointegrating equation with a significant level, indicating a long-term equilibrium relationship between the variables. Table 2 presents the eigenvalues and trace test statistics for four hypotheses. These hypotheses involve ranks of ≤3, ≤2, ≤1, and =0. The greatest value obtained from these tests is 0.477. The following column displays the trace test statistics alongside crucial values corresponding to specific confidence levels: 10%, 5%, and 1%. Applying the conventional method of using a 5% critical value, with ‘r’ denoting the rank and ‘r = 0’, the test statistics of 78.2 is greater than 53.1. Subsequently, null hypotheses that propose ‘r > 0’, which implies the existence of cointegration was rejected. When the value of r is less than or equal to 1, the test statistics of 33.44 is less than 34.9, the null hypotheses was not rejected. Ultimately, there is at most one cointegration relationship that exists at a significant level, elucidating a long-term state of balance among the variables.

**Table 2:** Johansen cointegration test results.

Rank,r	Eigenvalue	Trace statistics	10% Critical value	5% Critical value	1% Critical value
r=0	0.477	78.21	49.65	53.12	60.16
r≤1	0.208	33.44	32	34.91	41.07
r≤2	0.141	17.37	17.85	19.96	24.6
r≤3	0.095	6.86	7.52	9.24	12.97

The rank of the matrix A, is given by r and the Johansen test sequentially tests whether this rank r is equal to zero, equal to one, through to r = n − 1, where n is the number of time series under test. Eigenvectors and eigenvalues are fundamental concepts in linear algebra. The basic equation is Ax = λx. The number λ is an eigenvalue of A. The eigenvalue λ tells whether the special vector x is stretched or shrunk or reversed or left unchanged—when it is multiplied by A. The results include trace statistics and maximum eigenvalue statistics to determine the number of cointegrating relationships.

#### Cointegration vector.

Cointegrating vector equilibrium equation represents a long-term equilibrium relationship between the variables case, temperature, ovitrap index (OI), and sticky ovitrap index (SOI) as described in Table 3. The equation can be written as: Case−0.9507×Temperature+0.9566×OI−1.3755×SOI=0.

**Table 3:** Cointegrating Vector Equilibrium.

Variable	Coefficient
CASE	1.0000
TEMPERATURE	-0.9507
OVITRAP, OI	0.9566
S. OVITRAP, SOI	-1.3755

Interpreting the Coefficients are as follows;

- Case (1.0000): This means the variable case is normalized with a coefficient of 1, making it the dependent variable in this equilibrium relationship.
- Temperature (-0.9507): A negative coefficient means that as temperature increases by a unit, the number of dengue cases decrease by 0.9507 in the long run.
- Ovitrap index, OI (0.9566): A positive coefficient means that as the OI (which measures mosquito breeding activity) increases by a unit, dengue cases increase by 0.9566.
- Sticky ovitrap index, SOI (-1.3755): A negative coefficient suggests that as SOI increases by a unit, dengue cases decrease by 1.3755. This could indicate that SOI represents a control measure that helps reduce cases.

Over the long term, there is an inverse interaction between temperature and the sticky ovitrap index (SOI) with dengue cases, as compared to the ovitrap index (OI). This informative equilibrium provides us with the information that; persistence increment of temperature leads to decreases in dengue cases. The consistent and efficient sticky ovitraps, which capture adult mosquitoes over a lengthy period of time, have resulted in a decrease in dengue incidence. A consistently positive correlation between the coefficient of OI and dengue cases validates that OI can effectively serve as a reliable estimate of the *Aedes* population in areas with a high burden of dengue.

#### VECM Estimation.

When cointegration data series are present, the VECM model is used to calculate and include error correction terms that take into account any deviations from the long-term equilibrium. This model is well-suited for examining the short-term interactions between variables. A lag of p - 1 was included to adjust for the degrees of freedom used by the lag inclusion. Here is the VECM equation for this analysis:


ΔCASEt=λ+θ×ECTt−9+δ1ΔTEMPERATUREt−9+δ2ΔOIt−9+δ3ΔSOIt−9+ϵt

- Δ denotes the first difference of a variable.
- $ECT_{t-9}$ (Error Correction Term) is the lagged value of the deviation from the
- long-term equilibrium (residuals from the cointegration equation), included to bring the variables back towards equilibrium.
- γ, θ, δ_1_, δ_2_, δ_3_ are parameters to be estimated.
- ∊_t_ is the error term.

The model estimation requires the determination of 152 slope parameters, which illustrates the intricate and strong nature of the model in accurately representing the changes over various time intervals and interactions. The Akaike Information Criterion (AIC) and the Bayesian Information Criterion (BIC) quantify the goodness of fit and complexity of the model. The AIC has a value of 869.1324, while the BIC has a value of 1213.156. These criteria are useful in evaluating the model’s efficacy which is the trade-off between accuracy and complexity. This document elucidates the manner in which the variables being examined establish a connection within the equation. A model may exhibit exceptional fit or accuracy, although it lacks the capacity to elucidate the intricacies of variables. The greater the complexity of the dynamic, the lower the fit. In addition, the sum of squared residuals (SSR) is reported as 5510.52. This value serves as the total squared deviations of observed values from their predicted values within the model framework also known as a measure of the model’s overall error magnitude. Furthermore, it can be inferred that the model estimation has the capacity to elucidate 74.2% of the dynamics.

The VECM analysis shown in Table 4 offers a comprehensive understanding of the short-term interactions among dengue cases, temperature, and mosquito indices (ovitrap and sticky ovitrap) throughout time. The results reveal significant coefficients, especially in the error correction term and several lagged variables, demonstrating the impact of previous values on current trends in the data. The key interactions are as described;

**Table 4:** Significant coefficients in VECM analysis.

Equation	Variable	Coefficient (Standard Error)	Significance
CASE	ECT	-0.258 (0.065)	***
S. OVITRAP	S. OVITRAP -1	-0.713 (0.197)	**
CASE	CASE -3	0.358 (0.124)	**
CASE	S. OVITRAP -3	-1.164 (0.387)	**
CASE	S. OVITRAP -4	-1.455 (0.404)	**
OVITRAP	OVITRAP -4	-0.637(0.231)	**
CASE	TEMPERATURE -6	-7.511 (2.419)	**
CASE	CASE -7	0.417 (0.148)	**
CASE	CASE -8	0.411 (0.132)	**
S. OVITRAP	CASE -1	0.193 (0.08)	*
OVITRAP	OVITRAP -1	-0.436 (0.184)	*
OVITRAP	OVITRAP -3	-0.524 (0.213)	*
S. OVITRAP	S. OVITRAP -6	0.172(0.076)	*
S. OVITRAP	S. OVITRAP -8	2.885(1.4)	*
OVITRAP	OVITRAP -9	0.534(0.237)	*

Significance levels are denoted as *** (p <.001), ** (p <.01), and * (p <.05).

i. Error correction term (ECT) and case.

With ECT Coefficient of -0.258, the error correction term tells us how quickly dengue cases adjust to the long-term equilibrium. It indicates deviation from the long-term equilibrium, will be corrected around 25.8% in each period. Since the coefficient is negative, it means that when dengue cases deviate from their long-term trend, they slowly return to balance and the significance of this adjustment is statistically strong. This highlights the *efficacy of the dynamic interaction* which enable it to revert to its equilibrium within the model estimated.

ii. The interaction within sticky ovitrap equation with sticky ovitrap variable at lag-1.

It shows that the coefficient of 0.713 past values of sticky ovitrap (one period ago) negatively impact its current value. A coefficient of -0.713 means that for every 1-unit increase in sticky ovitrap in the previous period, the current value of sticky ovitrap decreases by 0.713 units. A negative coefficient means that if sticky ovitrap was high in the previous of one period ago, it tends to decrease in the current period significantly.

iii. The interaction within dengue case equation with dengue case variable at lag-3.

The positive coefficient (0.358) means that dengue cases from 3 periods ago have a direct positive impact on current dengue cases. 0.358 means that for every 1-unit increase in dengue cases three periods ago, the current number of cases increases by 0.358 units significantly.

**Fig 4:**
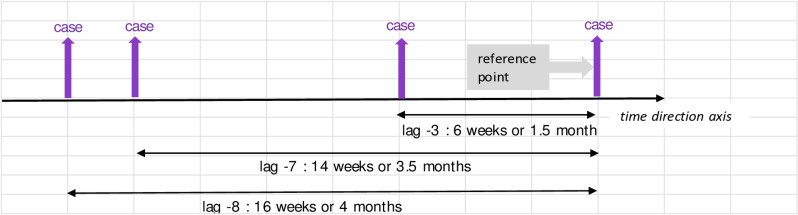
VECM output on short term dynamic where each lag denotes 2 weeks length of time. Interaction between case equation and case variable shows positive coefficient at lag -3, -7, -8.

**Fig 5:**
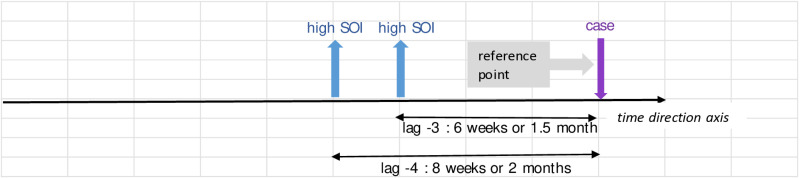
VECM output on short term dynamic where each lag denotes 2 weeks length of time. Interaction between case equation and SOI variable with negative coefficient at lag -3 and -4.

**Fig 6:**
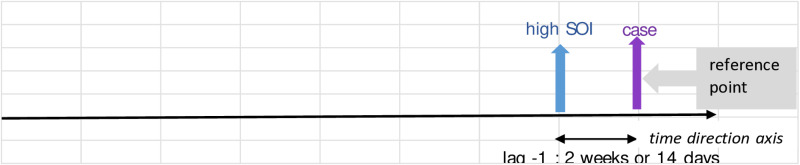
VECM output on short term dynamic where each lag denotes 2 weeks length of time. The interaction shows positive coefficient at lag -1.

**Fig 7:**
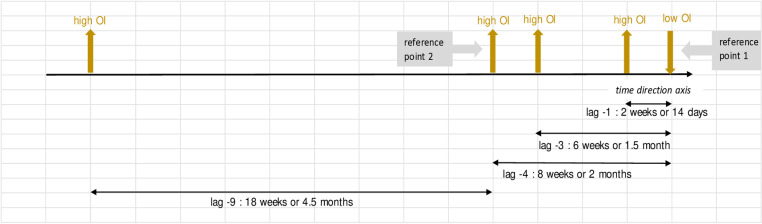
VECM output on short term dynamic where each lag denotes 2 weeks length of time. The result shows negative coefficients on the OI variable at lags -1, -3, and -4 and positive coefficient at lag -9.

**Fig 8:**
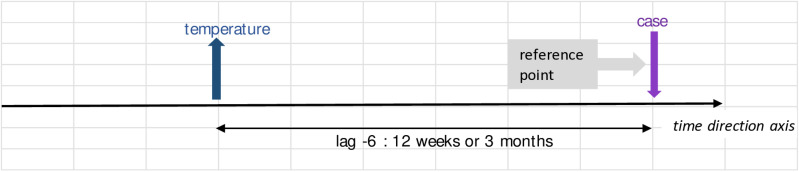
VECM output on short term dynamic where each lag denotes 2 weeks length of time. The interaction shows negative coefficient at lag -6.

**Fig 9:**
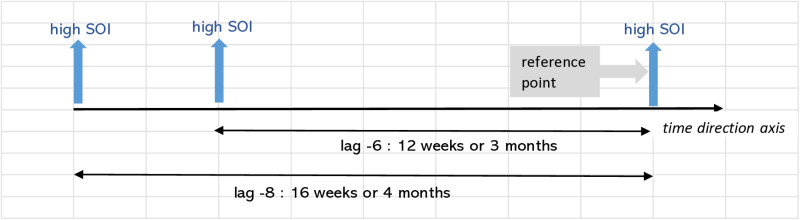
VECM output on short term dynamic where each lag denotes 2 weeks length of time. Interaction between SOI equation and SOI variable with positive coefficient at lag -6 and -8.

**Fig 10:**
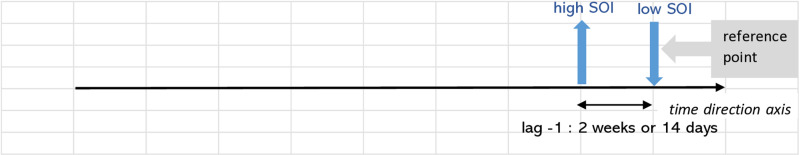
VECM output on short term dynamic where each lag denotes 2 weeks length of time. The interaction shows negative coefficient at lag -1.

Further interpretation of the values in Table 4 goes the same for all. These are also best described in illustration in fig 4 - 10; the VECM output on short term dynamic where each lag denotes 2 weeks length of time (fig 4–10).

For fig 4, the interaction between case equation and case variable denotes positive coefficient at lag -3, -7, -8. It highlights that preceding dengue cases have a continuing impact on future incidences. This allows us to understand that even though the transmission of dengue follows a cyclical pattern in the long term, it is affected by outbreaks, which cause deviations or fluctuations from a stable state. Consequently, this results in peaks in dengue cases or epidemics.

For fig 5, in the case equation with relation to SO variable at lag-3 and -4 shows negative coefficients. It reveals that effective *Aedes* control underscoring the effectiveness of these tool as interventions over time. ^It^ reveals the latent effect (1.5–2 months later) of the sticky ovitrap tool as a control measure impacting dengue cases.

For fig 6, this indicates that SOI acts as a surveillance tool with plausible aligning that transmission occurs within the next 14 days. Hence, an increase in adult *Aedes* mosquito populations will result in the transmission of dengue fever and development of cases.

For fig 7, interaction between OI equation and OI variable with negative coefficients on the OI variable at lags -1, -3, and -4 suggest a rapid (brief period of time) external influence resulting in the decline of *Aedes* larvae in a time frame of two weeks up to two months, likely due to prompt control measures or other interacting factors. At lag -9, with positive coefficient on the OI variable indicates that the population of larvae will ultimately increase later (latency -9 or 4.5 months later), as a result of inability to maintain control measures or other interactions that arise within the ecological system.

For fig 8, the significant negative coefficient of temperature at lag-6 for case equation, shows the interplay of persistent increasing of temperature within the ecological link. This temperature divergance from its stable state will influence dengue instances three months later, leading to decline of dengue cases subsequently.

For fig 9 & 10, the positive coefficients of sticky ovitrap index, SOI, in the sticky ovitrap equation at lags -6 and -8 demonstrated the sustained effectiveness of sticky traps. This indicates the crucial use of these tools as part of strategy in persistently high populations of the *Aedes* mosquito. This discovery suggests that the current existence of adult *Aedes* mosquitoes will have a substantial impact on the population’s growth after 6 and 8 biweekly intervals, corresponding to 3 and 4 months, respectively. However, at a lag of -1 (a gap of 14 days) and a negative coefficient, a low current capture measurement of SOI is associated with a past high SOI reading. This explains the ecological dynamics of *Aedes* mosquitoes and their life cycle, which includes the duration of the incubation period of the dengue virus. The dengue virus undergoes an incubation period of around 7–10 days inside the mosquito following its ingestion of blood from an infected individual. This explains that the particular phase of the life cycle is very much related to temporal fluctuations of the mosquito population and the impact on the utilization of sticky ovitraps within the ecological system.

#### Forecasting based on VECM.

**Fig 11:**
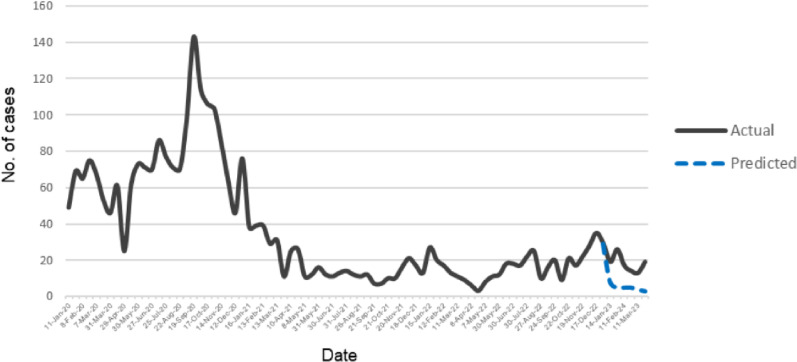
Actual case count line graph from January 2020 to March 2023 and predicted case count line graph of dengue case plotted from January 2023 to March 2023, extracted from R.

In the evaluation of the Vector Error Correction Model (VECM) for forecasting purposes, the forecasted biweekly onset values were meticulously compared against the biweekly average actual values observed over a 12-week period, commencing from January 2023 until March 2023 (Fig 11).

A comparative analysis is carried out to assess the model’s predictive accuracy (Table 5) and reliability in estimating real-world occurrences within the specified timeframe.

**Table 5:** Statistical metric values.

Metric	Value
MSE	176.2
RMSE	13.28
MAPE	2.72%

The three main statistical metrics used to measure the differences between the VECM predictions and the actual observed values were the mean squared error (MSE); the root mean square error (RMSE) and a third metric known as the mean absolute percentage error (MAPE).

The MSE measures the variance of errors (squared differences between actual and predicted values). While a lower MSE indicates greater accuracy, its interpretation is dependent on the scale of the data. Here, 176.42 indicates a moderate level of error dispersion, but its importance should be evaluated in comparison to other models on the same dataset, if any.

The RMSE, which is generated from MSE, gives a simple measure of the model’s typical forecasting error in the same units as the original data.

The MAPE represents the average percentage deviation of predicted values from actual values. A MAPE of 2.72% indicates that, on average, the forecasted values are very close to actual values, with a small percentage error. In general:

◦MAPE < 10% → Highly accurate forecast
◦10% ≤ MAPE < 20% → Good forecast
◦20% ≤ MAPE < 50% → Reasonable forecast
◦MAPE ≥ 50% → Poor forecast

## Discussion

VECM, a multivariate regression time series analysis chosen, is able to establish both the long-term and short-term deviations among variables dynamicity. The study method in general was previously commonly used in economic studies to explain dynamic interaction among variables [25–27]. It is able to reduce cofounding factors, demonstrates variable cointegration, captures the causal effect either directly or inversely, and can be utilised for further forecasting. Therefore, *VECM plays a pivotal role in understanding the past to guide the future and is not just about predicting the future alone*.

This predictive performance incorporating three metrics are useful in delivering the relative accuracy of the forecasts, allowing stakeholders to look into the potential impact of forecast errors in operational terms [28,29]. The overall model performance with low RMSE (13.28) and minimal MAPE (2.72%) suggest high accuracy and reliable forecasting performance. The MSE (176.42), indicates that the model’s error variance is reasonable. The performance evaluation considers the data’s characteristics (i.e.,; stationarity, sample size and seasonality) and the stability of error metrics accounted. Therefore, making it well-suited for time series forecasting in this study’s context. The study cannot be readily compared with other time series studies due to its distinctive methodology and contextual factors including the data characteristics.

In the context of public health and environmental health, it is still a new methodology to capture such dynamicity and causality. Knowledge on all the variables studied is essential to estimating the best fit model equation and yet being able to provide plausible interaction complexity at the same point. The key interactions highlighted are;

i. Dengue cases

In long term interaction, the study expressed the temporal pattern of dengue transmission, which is persistent, stable, and cyclical in nature. Short-term deviations from equilibrium in the form of peaks of dengue cases or epidemics are influenced by past outbreaks.

ii. Sticky ovitrap index

Consistent and efficient sticky ovitraps installation has resulted in a decrease in cases long-term. Short-term equilibrium shows that sticky ovitrap plays a dual role, acting as a surveillance tool at lag-1 (within 14 days) and as a control tool at lag-3 and -4, which is the latent effect (1.5–2 months later) impacting dengue cases.

iii. Ovitrap index

The long-term equilibrium indicates the ovitrap index as a reliable predictor of dengue cases in an area with a significant dengue burden and a reliable marker for larval density short-term. In addition, consistently high current OI readings in an area will determine the persistence of the larval population between four and five months later.

iv. Temperature

Temperature has indirect impact on dengue cases but its interaction pathway involving all the three variables is multifaceted and complex. Temporary divergence, referring to a variation in temperature from its stable state, will influence dengue instances three months later. Nevertheless, the temperature has shown a zero-lag association with sticky ovitrap and ovitrap index. The ecological system interplays may be attributed to the dynamicity of the relationship between temperature and dengue cases, as it still remains unclear on the complexity of the interaction, namely the vector transmission and vector competence.

The implication of this research is that it offers a valid agreement for the execution of a successful dengue epidemic plan. Public health officers need to manage outbreaks efficiently and treat them specifically, as each outbreak is unique in nature. Each ‘Incident Action Plan’ (IAP) of the outbreak implemented must address the main issues with the goal of preventing the progression of succession and rapid spread of dengue cases. In addition, emphasising the need for historical data consideration and continuous analysis would be helpful for future planning. Intriguingly, many countries in Southeast Asia exhibit a cyclical trend, experiencing surges in dengue infections throughout specific periods. The apparent high prevalence of dengue and amplification of prior outbreaks in the Southeast Asia region can be partly attributed to hyperendemicity, which refers to the simultaneous circulation of all four serotypes of the virus [30,31]. This corresponds to the model interaction dynamics, in parallel with the surge in the number of cases of the current outbreak and is proven to be connected to prior dengue cases.

The utilisation of the sticky ovitraps within a short period of time of 14 days demonstrates the interplay between the bionomics and life cycle of *Aedes*. The tool acts primarily as a surveillance tool rather than a control tool. It’s an indicator for ongoing rapid and continuous transmission of the dengue virus concurrently. Consequently, prompt control methods are needed in conjunction with the use of sticky traps. It also implies subsequent measures or evaluations in the area affected by dengue are crucial after implementing an initial control intervention or first control cycle.

Crucially, the outcome also shows how the “latent effect” of installing sticky ovitraps as a preventative step affects dengue cases. Therefore, a thorough action plan must include the subsequent measures. Consistent deployment of sticky ovitraps in an area with a high prevalence of dengue fever has proven to have a direct impact on occurrences of dengue cases. It showcased its utility and efficacy as a component of a control approach.

Recently, the conventional approach of employing insecticides to manage adult female *Aedes* mosquitoes has been replaced by a new approach that focuses on the creation and evaluation of non-insecticidal methods [32,33]. The increasing apprehension regarding the development of pesticide resistance and its adverse effects on the environment and human health is a significant catalyst for the adoption of safer and more sustainable technologies [34]. In Malaysia, rising pesticide resistance in *Aedes* mosquito populations seemed to compromise dengue control efforts. Studies in Malaysia found resistance to common pesticides in both *Aedes aegypti* and *Aedes albopictus* mosquitos. This resistance poses a substantial threat to current vector control tactics, highlighting the critical need for coordinated resistance management in the National Dengue Control Program [35,36].

With that, sticky ovitrap has been frequently used in the monitoring and controlling of adult mosquitoes [33,37,38]. Enhancing the control efforts with the utilisation of mass trapping of sticky ovitraps to control adult *Aedes* populations has shown encouraging effectiveness recently [38,39]. Nevertheless, the level of certainty regarding these findings is only moderate. Several key elements are missing, such as the study design, the operationalisation, the standard index used, and the analysis of the data, including the outcome or epidemiological endpoint [38–40]. Indeed, the outcomes of this model’s dynamic analysis will unequivocally demonstrate the efficacy of sticky ovitraps intervention in complementing measures to mitigate dengue cases. It offers a quicker and more direct assessment of the efficacy of ovipositional attractants while also catching the carrier of circulating Den V.

Therefore, a crucial implication of this study is to explore protocols for operational deployment circumstances in order to identify the best deployment parameters under various epidemiological scenarios. It is imperative to take into account the guidelines of the World Health Organization and adapt them to the specific operational conditions of the control team [40]. Addressing a logistical and financial investment setback in the trap’s deployment is crucial. In conjunction with that, cost-effectiveness and cost-benefit should be carried out as part of an economic evaluation measure. In conjunction with the 3rd and 11th Sustainable Development Goals (SDGs) [41], under sustainable engagement circumstances, the expansion of installing sticky ovitraps would be a priority for *urban areas where adulticide control is not feasible*, such as exclusion zones, proximity to water basins, hospitals, insectariums, *areas of possible insecticide resistance*, and *a community-based intervention*.

For the second entomological variable (OI), this study validated the role of ovitrap installation as part of vector surveillance tool. It is a monitoring tool for larval density that can trigger appropriate control measures. Apart from that, it is also recommended to be used as a marker for exit strategy after a certain time frame prior to relocating the tool to another site in countries or regions with limited resources. A recent forecasting study conducted in Brazil, employing a different analytical model, corroborates this claim by indicating that the utilization of ovitraps has the potential to identify dengue epidemics with a lead time of four to six weeks [30].

The dynamic pathway of temperature effect on dengue cases have been unclear for decades. The mechanism is thought to be in relation with biological characteristics of *Aedes*, vector competence and direct impact on circulating Den virus within the mosquitoes [42]. Mosquitoes are poikilothermic organisms, meaning that variations in the ambient temperature have an immediate impact on their internal body temperature. Although warmer temperatures can initially facilitate mosquito breeding and virus transmission, they can also result in long-term ecological adaptations that help control mosquito populations. These adaptations may include greater predator activity or faster virus incubation, which can lead to early viral die-offs [42,43], a shortened gonotrophic cycle [44,45], and later a significant reduction in the number of eggs produced, resulting in a decrease in the size of the *Aedes* population [46–48].

In relation to vector competence, the transmission of the virus is diminished when the temperature exceeds 32 °C, resulting in a decrease in its ability to be transmitted by its carrier [49,50]. This is intimately related to immune system pathways and tissue barriers in the mosquito, which lower the rate of midgut infections and DEN V transmission [51]. DEN V is a single-positive-stranded RNA virus that encodes three structural proteins and seven non-structural proteins. The temperature-induced structural alterations usually occur between 31°C and 35°C and are irreversible, weakening the E protein [52,53]. Hence, it diminishes viral infectivity. In general, it is imperative to thoroughly examine the impact of global warming on the survival of *Aedes* mosquitoes. This will certainly interfere with the transmission of dengue fever. The impact on warmer climate regions results in being closer to the thermal thresholds that many different kinds of species have. It is also necessary to consider the impact on cooler regions, which encourages the spread of vector borne illnesses [54]. Therefore, it is essential to closely monitor this climate change signal and how it affects the adaptability of the vector.

In summary of the preceding findings, previous dengue occurrences predicted a surge of new dengue cases while preserving the cyclical and continuous pattern. The model predicts the utility and efficacy of sticky ovitrap, thereby establishing and reinforcing its dual roles in aiding strategy. It is a turning point for control measures to consider heavily on the application of mass sticky ovitrapping as part of integrated vector management. The result also reinforced the use of ovitrap as a surveillance tool and offered substantiation of the influence of temperature on the progression of dengue cases. Ultimately, refining these results into strategies and policies will enhance public health responses progressively to vector-borne diseases.

Temperature is indeed an overall excellent estimator of the meteorological parameter that has a direct impact on the development of dengue cases. It is recommended to use more extensive data to look into its temporal interaction with ovitrap, sticky ovitrap findings, and progression of virus in mosquitoes.

## Limitation

This study does not consider variables such as demography, population immunity, mosquito virus surveillance, or the socio-economic structure of society. Historically, there has been a lack of consistent collection of serial data on virus surveillance in mosquitoes. Incorporating the virus surveillance in mosquitoes or also known as ‘virus detection in mosquitoes’, VDIM variable may contribute to the demonstration of the complex interaction. This study also recognizes the significance of vector control in the potential disruption of equilibrium. This study focused exclusively on dengue cases that were diagnosed and reported solely to the health district. Hence, the system’s ability to accurately document the overall number of dengue cases may be compromised due to the presence of diverse dengue symptoms, resulting in instances where illnesses go unnoticed or undetected.

## References

[1] Gubler DJ. Dengue, urbanization and globalization: the unholy trinity of the 21st Century. Trop Med Health; 2011. 10.2149/tmh.2011-S05 PMC3317603 22500131

[2] World Health Organization. Dengue guidelines for diagnosis, treatment, prevention and control: new edition. World Health Organization. 2009 [cited 2024 May 20]. Available from: https://apps.who.int/iris/handle/10665/44188 23762963

[3] Morrison AC, Zielinski-Gutierrez E, Scott TW, Rosenberg R. Defining challenges and proposing solutions for control of the virus vector Aedes aegypti. PLoS Med. 2008;5(3):e68. doi: 10.1371/journal.pmed.0050068 18351798 PMC2267811

[4] Djiappi-Tchamen B, Nana-Ndjangwo MS, Nchoutpouen E, Makoudjou I, Ngangue-Siewe IN, Talipouo A, et al. Aedes Mosquito Surveillance Using Ovitraps, Sweep Nets, and Biogent Traps in the City of Yaoundé, Cameroon. Insects. 2022;13(9):793. doi: 10.3390/insects13090793 36135494 PMC9500714

[5] Fountuora G. Knowledge discovery and dengue forecasting applied in a four-year dataset collected at Natal/RN – Brazil. Environmental Science Medicine. 2021; doi: 10.21203/rs.3.rs-955820/v1

[6] de Albuquerque BC, Pinto RC, Sadahiro M, Sampaio VS, de Castro DB, Terrazas WCM, et al. Relationship between local presence and density of Aedes aegypti eggs with dengue cases: a spatial analysis approach. Trop Med Int Health. 2018;23(11):1269–79. doi: 10.1111/tmi.13150 30282110

[7] Fansiri T, Buddhari D, Pathawong N, Pongsiri A, Klungthong C, Iamsirithaworn S, et al. Entomological Risk Assessment for Dengue Virus Transmission during 2016-2020 in Kamphaeng Phet, Thailand. Pathogens. 2021;10(10):1234. doi: 10.3390/pathogens10101234 34684183 PMC8538081

[8] Oliveira Noleto JV, Moura do Nascimento Moraes HL, De Moura Lima T, Mendes Rodrigues JG, Tavares Cardoso D, Chaves Lima K, et al. Use of ovitraps for the seasonal and spatial monitoring of Aedes spp. in an area endemic for arboviruses in Northeast Brazil. J Infect Dev Ctries. 2020;14(4):387–93. doi: 10.3855/jidc.12245 32379716

[9] Resende MC de, Silva IM, Ellis BR, Eiras ÁE. A comparison of larval, ovitrap and MosquiTRAP surveillance for Aedes (Stegomyia) aegypti. Mem Inst Oswaldo Cruz. 2013;108(8):1024–30. doi: 10.1590/0074-0276130128 24402144 PMC4005541

[10] Patil S, Pandya S. Forecasting Dengue Hotspots Associated With Variation in Meteorological Parameters Using Regression and Time Series Models. Front Public Health. 2021;9:798034. doi: 10.3389/fpubh.2021.798034 34900929 PMC8661059

[11] Lowe R, Gasparrini A, Van Meerbeeck CJ, Lippi CA, Mahon R, Trotman AR, et al. Nonlinear and delayed impacts of climate on dengue risk in Barbados: A modelling study. PLoS Med. 2018;15(7):e1002613. doi: 10.1371/journal.pmed.1002613 30016319 PMC6049902

[12] Mordecai EA, Caldwell JM, Grossman MK, Lippi CA, Johnson LR, Neira M, et al. Thermal biology of mosquito-borne disease. Ecol Lett. 2019;22(10):1690–708. doi: 10.1111/ele.13335 31286630 PMC6744319

[13] Liyanage P, Tissera H, Sewe M, Quam M, Amarasinghe A, Palihawadana P, et al. A Spatial Hierarchical Analysis of the Temporal Influences of the El Niño-Southern Oscillation and Weather on Dengue in Kalutara District, Sri Lanka. Int J Environ Res Public Health. 2016;13(11):1087. doi: 10.3390/ijerph13111087 27827943 PMC5129297

[14] Lefebvre B, Karki R, Misslin R, Nakhapakorn K, Daudé E, Paul RE. Importance of Public Transport Networks for Reconciling the Spatial Distribution of Dengue and the Association of Socio-Economic Factors with Dengue Risk in Bangkok, Thailand. Int J Environ Res Public Health. 2022;19(16):10123. doi: 10.3390/ijerph191610123 36011755 PMC9408777

[15] Singh S, Herng LC, Sulaiman LH, Wong SF, Jelip J, Mokhtar N, et al. The Effects of Meteorological Factors on Dengue Cases in Malaysia. Int J Environ Res Public Health. 2022;19(11):6449. doi: 10.3390/ijerph19116449 35682035 PMC9180499

[16] Rohani A, Suzilah I, Malinda M, Anuar I, Mohd Mazlan I, Salmah Maszaitun M, et al. Aedes larval population dynamics and risk for dengue epidemics in Malaysia. Trop Biomed. 2011;28(2):237–48. 22041742

[17] Ahmad R, Suzilah I, Wan Najdah WMA, Topek O, Mustafakamal I, Lee HL. Factors determining dengue outbreak in Malaysia. PLoS One. 2018;13(2):e0193326. doi: 10.1371/journal.pone.0193326 29474401 PMC5825112

[18] Ismail S, Fildes R, Ahmad R, Wan Mohamad Ali WN, Omar T. The practicality of Malaysia dengue outbreak forecasting model as an early warning system. Infect Dis Model. 2022;7(3):510–25. doi: 10.1016/j.idm.2022.07.008 36091345 PMC9418377

[19] Melaka Health Department. Vectorborne disease annual report; 2023.

[20] Arunachalam N, Samuel P, Hiriyan J, Gajanana A. A comparative study on sampling techniques for Aedes aegypti (diptera: culicidae) surveillance in Madurai, South India. Tropical Biomedicine. 1999;16:25–9.

[21] Richards SL, Apperson CS, Ghosh SK, Cheshire HM, Zeichner BC. Spatial analysis of Aedes albopictus (Diptera: Culicidae) oviposition in suburban neighborhoods of a Piedmont community in North Carolina. J Med Entomol. 2006;43(5):976–89. doi: 10.1603/0022-2585(2006)43[976:saoaad]2.0.co;2 17017237

[22] Ritchie SA, Long S, Smith G, Pyke A, Knox TB. Entomological investigations in a focus of dengue transmission in Cairns, Queensland, Australia, by using the sticky ovitraps. J Med Entomol. 2004;41(1):1–4. doi: 10.1603/0022-2585-41.1.1 14989339

[23] Lütkepohl H. New introduction to multiple time series analysis. Berlin: Springer. 2005.

[24] Wee P, Tan R. Performance of Johansen’s cointegration test. In East Asian Economic Issues. 1997;3.

[25] Peleshchak R, et al. A vector error correction model approach to analyze the causality among SME export-import activity and the economic development of EU countries. 8th International Conference on Computational Linguistics and Intelligent Systems. 2024.

[26] Zhiheng Y. The relationship between consumption and economic growth of Chinese urban and rural residents since reform and opening-up -- an empirical analysis based on econometrics models. General Economics. 2022. doi: 10.2305/02138

[27] Dominique NN, Buntaran CII, Nurhanifah A, Ferdinand FV. G20 Economic Growth Analysis Using VECM. JIETcon. 2023;8(2):338–59. doi: 10.20473/jiet.v8i2.50361

[28] Hyndman RJ, Koehler AB. Another look at measures of forecast accuracy. International Journal of Forecasting. 2006;22(4):679–88. doi: 10.1016/j.ijforecast.2006.03.001

[29] Makridakis S, Wheelwright SC, Hyndman RJ. Forecasting: methods and applications. John Wiley & Sons. 1998.

[30] Cattarino L, Rodriguez-Barraquer I, Imai N. Mapping global variation in dengue transmission intensity. Sci Transl Med. 2020; 12:eaax4144. 31996463 10.1126/scitranslmed.aax4144

[31] Lim JT, Dickens BS, Tan KW, Koo JR, Seah A, Ho SH, et al. Hyperendemicity associated with increased dengue burden. J R Soc Interface. 2021;18(182):20210565. doi: 10.1098/rsif.2021.0565 34520691 PMC8440027

[32] Jaffal A, Fite J, Baldet T, Delaunay P, Jourdain F, Mora-Castillo R, et al. Current evidences of the efficacy of mosquito mass-trapping interventions to reduce Aedes aegypti and Aedes albopictus populations and Aedes-borne virus transmission. PLoS Negl Trop Dis. 2023;17(3):e0011153. doi: 10.1371/journal.pntd.0011153 36877728 PMC10032496

[33] Barrera R. New tools for Aedes control: mass trapping. Curr Opin Insect Sci. 2022;52:100942. doi: 10.1016/j.cois.2022.100942 35667560 PMC9413017

[34] European Environment Agency. How pesticides impact human health and ecosystems in Europe. 2023. Available from: https://www.eea.europa.eu/publications/how-pesticides-impact-human-health/how-pesticides-impact-human-health

[35] Rasli R, Cheong YL, Che Ibrahim MK, Farahininajua Fikri SF, Norzali RN, Nazarudin NA, et al. Insecticide resistance in dengue vectors from hotspots in Selangor, Malaysia. PLoS Negl Trop Dis. 2021;15(3):e0009205. doi: 10.1371/journal.pntd.0009205 33755661 PMC7987141

[36] Ishak IH, Jaal Z, Ranson H, Wondji CS. Contrasting patterns of insecticide resistance and knockdown resistance (kdr) in the dengue vectors Aedes aegypti and Aedes albopictus from Malaysia. Parasit Vectors. 2015;8:181. doi: 10.1186/s13071-015-0797-2 25888775 PMC4377062

[37] Johnson BJ, Ritchie SA, Fonseca DM. The State of the Art of Lethal Oviposition Trap-Based Mass Interventions for Arboviral Control. Insects. 2017;8(1):5. doi: 10.3390/insects8010005 28075354 PMC5371933

[38] Englbrecht C, Gordon S, Venturelli C, Rose A, Geier M. Evaluation of BG-Sentinel Trap as a Management Tool to Reduce Aedes albopictus Nuisance in an Urban Environment in Italy. J Am Mosq Control Assoc. 2015;31(1):16–25. doi: 10.2987/14-6444.1 25843172

[39] Degener CM, Eiras AE, Azara TMF, Roque RA, Rösner S, Codeço CT, et al. Evaluation of the effectiveness of mass trapping with BG-sentinel traps for dengue vector control: a cluster randomized controlled trial in Manaus, Brazil. J Med Entomol. 2014;51(2):408–20. doi: 10.1603/me13107 24724291

[40] World Health Organization. Efficacy-testing of traps for control of aedes spp. mosquito vectors. WHO Geneva. Contract No.: WHO/CDS/NTD/VEM/2018.06. 2018.

[41] United Nations. Department of Economic and Social Affairs. Sustainable Development. The 17 Goals | Sustainable Development; 2023.

[42] Morin CW, Comrie AC, Ernst K. Climate and dengue transmission: evidence and implications. Environ Health Perspect. 2013;121(11–12):1264–72. doi: 10.1289/ehp.1306556 24058050 PMC3855512

[43] Alto BW, Bettinardi D. Temperature and dengue virus infection in mosquitoes: independent effects on the immature and adult stages. Am J Trop Med Hyg. 2013;88(3):497–505. doi: 10.4269/ajtmh.12-0421 23382163 PMC3592531

[44] Delatte H, Gimonneau G, Triboire A, Fontenille D. Influence of temperature on immature development, survival, longevity, fecundity, and gonotrophic cycles of Aedes albopictus, vector of chikungunya and dengue in the Indian Ocean. J Med Entomol. 2009;46(1):33–41. doi: 10.1603/033.046.0105 19198515

[45] Carrington LB, Armijos MV, Lambrechts L, Barker CM, Scott TW. Effects of fluctuating daily temperatures at critical thermal extremes on Aedes aegypti life-history traits. PLoS One. 2013;8(3):e58824. doi: 10.1371/journal.pone.0058824 23520534 PMC3592833

[46] Myer MH, Fizer CM, Mcpherson KR, Neale AC, Pilant AN, Rodriguez A, et al. Mapping Aedes aegypti (Diptera: Culicidae) and Aedes albopictus Vector Mosquito Distribution in Brownsville, TX. J Med Entomol. 2020;57(1):231–40. doi: 10.1093/jme/tjz132 31400202 PMC6951034

[47] Iwamura T, Guzman-Holst A, Murray KA. Accelerating invasion potential of disease vector Aedes aegypti under climate change. Nat Commun. 2020;11(1):2130. doi: 10.1038/s41467-020-16010-4 32358588 PMC7195482

[48] Nik Abdull Halim NMH, Che Dom N, Dapari R, Salim H, Precha N. A systematic review and meta-analysis of the effects of temperature on the development and survival of the Aedes mosquito. Front Public Health. 2022;10:1074028. doi: 10.3389/fpubh.2022.1074028 36600940 PMC9806355

[49] Liu Z, Zhang Z, Lai Z, Zhou T, Jia Z, Gu J, et al. Temperature Increase Enhances Aedes albopictus Competence to Transmit Dengue Virus. Front Microbiol. 2017;8:2337. doi: 10.3389/fmicb.2017.02337 29250045 PMC5717519

[50] Ciota AT, Chin PA, Ehrbar DJ, Micieli MV, Fonseca DM, Kramer LD. Differential Effects of Temperature and Mosquito Genetics Determine Transmissibility of Arboviruses by Aedes aegypti in Argentina. Am J Trop Med Hyg. 2018;99(2):417–24. doi: 10.4269/ajtmh.18-0097 29869610 PMC6090362

[51] Liu Z, Zhang Q, Li L, He J, Guo J, Wang Z, et al. The effect of temperature on dengue virus transmission by Aedes mosquitoes. Front Cell Infect Microbiol. 2023;13:1242173. doi: 10.3389/fcimb.2023.1242173 37808907 PMC10552155

[52] Zhang X, Sheng J, Plevka P, Kuhn RJ, Diamond MS, Rossmann MG. Dengue structure differs at the temperatures of its human and mosquito hosts. Proc Natl Acad Sci U S A. 2013;110(17):6795–9. doi: 10.1073/pnas.1304300110 23569243 PMC3637732

[53] Sharma KK, Lim X-X, Tantirimudalige SN, Gupta A, Marzinek JK, Holdbrook D, et al. Infectivity of Dengue Virus Serotypes 1 and 2 Is Correlated with E-Protein Intrinsic Dynamics but Not to Envelope Conformations. Structure. 2019;27(4):618-630.e4. doi: 10.1016/j.str.2018.12.006 30686666

[54] Paz S. Climate change: A driver of increasing vector-borne disease transmission in non-endemic areas. PLoS Med. 2024;21(4):e1004382. doi: 10.1371/journal.pmed.1004382 38574178 PMC11025906

